# Tumor Necrosis Factor Alpha Effects on the Porcine Intestinal Epithelial Barrier Include Enhanced Expression of TNF Receptor 1

**DOI:** 10.3390/ijms22168746

**Published:** 2021-08-14

**Authors:** Linda Droessler, Valeria Cornelius, Alexander G. Markov, Salah Amasheh

**Affiliations:** 1 Institute of Veterinary Physiology, Department of Veterinary Medicine, Freie Universität Berlin, 14163 Berlin, Germany; linda.droessler@fu-berlin.de (L.D.); valeria.cornelius@fu-berlin.de (V.C.); 2 Department of General Physiology, Faculty of Biology, Saint Petersburg State University, Universitetskaya nab., 7–9, 199034 Saint Petersburg, Russia; a.markov@spbu.ru

**Keywords:** claudins, epithelial barrier, IPEC-J2, ML-7, tight junction, TNFα, TNFR-1

## Abstract

Tumor necrosis factor alpha (TNFα) has been shown to impair the intestinal barrier, inducing and maintaining inflammatory states of the intestine. The aim of the current study was to analyze functional, molecular and regulatory effects of TNFα in a newly established non-transformed jejunal enterocyte model, namely IPEC-J2 monolayers. Incubation with 1000 U/mL TNFα induced a marked decrease in transepithelial electrical resistance (TEER), and an increase in permeability for the paracellular flux marker [^3^H]-D-mannitol compared to controls. Immunoblots revealed a significant decrease in tight junction (TJ) proteins occludin, claudin-1 and claudin-3. Moreover, a dose-dependent increase in the TNF receptor (TNFR)-1 was detected, explaining the exponential nature of pro-inflammatory effects, while TNFR-2 remained unchanged. Recovery experiments revealed reversible effects after the removal of the cytokine, excluding apoptosis as a reason for the observed changes. Furthermore, TNFα signaling could be inhibited by the specific myosin light chain kinase (MLCK) blocker ML-7. Results of confocal laser scanning immunofluorescence microscopy were in accordance with all quantitative changes. This study explains the self-enhancing effects of TNFα mediated by MLCK, leading to a differential regulation of TJ proteins resulting in barrier impairment in the intestinal epithelium.

## 1. Introduction

The intestinal epithelial paracellular barrier is essential since it prevents the immune system from being overly induced by a wide variety of exogenous antigens and microorganisms. Tight junctions (TJs), which are formed in strands and located at the apicolateral membrane of adjacent cells, represent the functional correlate of the epithelial paracellular barrier function. These TJ strands are defined by the common presence of several integral membrane proteins, such as occludin [1], junctional adhesion molecule (JAM) [2] or the claudin protein family, from which at least 27 members are known [3]. Along the longitudinal axis of the gastrointestinal tract, a strong correlation of TJ protein expression and barrier properties has been demonstrated [4]. In inflammatory bowel disease (IBD), increased paracellular permeability due to a lack or disruption of TJ proteins results in intestinal inflammation and may cause diarrhea [5].

The proinflammatory cytokine tumor necrosis factor α (TNFα) plays a key role in the pathogenesis of IBD, and thus has been shown to be increased in patients with Crohn’s disease (CD) or ulcerative colitis (UC) [6,7]. One potential proinflammatory action of TNFα is the ability to alter the intestinal epithelial TJ composition, leading to an increase in paracellular permeability and a decrease in transepithelial electrical resistance (TEER) [8,9]. Sealing TJ proteins, such as claudin-1, -7 or occludin, have been reported to be a primary target affected by incubation with TNFα [10], and the molecular changes in TJ composition cause a disturbance in intestinal epithelial barrier function and provoke diarrhea [11,12]. Although the exact mechanisms of TNFα on TJ composition have not been analyzed in detail, the myosin light chain kinase (MLCK) appears to be involved in intestinal barrier disturbance, as increased MLCK production can be observed after TNFα treatment [13,14,15]. One function of MLCK is to phosphorylate the myosin light chain (MLC), resulting in a contraction of actin and therefore rearranging the cytoskeleton [16,17]. In several inflammatory diseases, such as IBD, an enhanced expression of MLCK has been observed, leading to impaired epithelial barrier function [16]. The activation of MLCK by TNFα leads to a reorganization of TJ proteins and therefore affects the paracellular barrier [18].

The permanent porcine intestinal epithelial cell line IPEC-J2, a non-transformed cell line obtained from jejunal epithelia of an unsuckled piglet, has been characterized as a suitable in vitro intestinal model [19]. Due to its capacity to grow as a monolayer and form an apical and a basolateral compartment, as well as apicolateral junctional complexes, it can be useful for the analysis of the paracellular barrier and permeability. By culturing this cell line under species-specific conditions, using porcine serum (PS) instead of conventional serum, IPEC-J2 cells constitute an improved model for the analysis of porcine jejunal barrier function in vitro [20]. On account of the great similarities between the anatomy and physiology of swine and humans, the pig provides an extremely valuable model for studies compared to other animal models [21], as it is in accordance with human nutritional and digestive effects and mechanisms [22].

The barrier-weakening effect of TNFα has been evaluated on various cell culture models, such as the rat ileal intestinal epithelial cell line IEC-18 [23] or the human carcinogenic cell lines Caco-2 [24,25] and HT-29/B6 [10,26,27], but not in detail on the non-transformed porcine intestinal epithelial cell line IPEC-J2 so far. Therefore, our study aimed to analyze the effect of TNFα on the barrier function as well as the TJ composition of IPEC-J2 cells. This was assessed by the measurement of TEER and [^3^H]-D-Mannitol flux during treatment with the cytokine. Subsequent to the incubation experiments, the TJ composition was analyzed in detail and the impact of TNFα on the density of tumor necrosis factor receptor 1 and 2 (TNFR-1, TNFR-2) was investigated. As MLCK has been discussed in context with barrier perturbation in a series of studies, further experiments were carried out using a specific blocker against MLCK to analyze the signaling cascade of TNFα on IPEC-J2 cells. This provides new experimental data, showing that (i) the non-transformed cell line IPEC-J2 represents a useful model for the exploration of inflammation-induced changes in porcine intestinal epithelial barrier function and (ii) explains the self-enhancing effects of TNFα resulting in TJ impairment in the intestinal epithelium on a functional, molecular and regulatory level.

## 2. Results

### 2.1. Effects of TNFα on Epithelial Barrier Function of IPEC-J2

**Figure 1:**
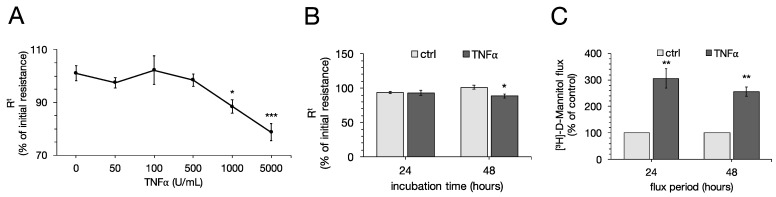
(**A**) To examine the dose-dependent effect of tumor necrosis factor α (TNFα) on IPEC-J2 cells, increasing concentrations were added basolateral to the monolayers and transepithelial electrical resistance (TEER) was measured for 48 h. Concentrations of 1000 U/mL and 5000 U/mL revealed a decrease in TEER in IPEC-J2 cells (one-way ANOVA, * *p* < 0.05, *** *p* < 0.001; *n* = 14–22). Therefore, following experiments were carried out using 1000 U/mL. (**B**) No significant changes in TEER could be observed after 24 h with 1000 U/mL TNFα. (**C**) Flux measurements using [^3^H]-D-Mannitol revealed an increased paracellular flux after 24 as well as 48 h (unpaired *t*-test, ** *p* < 0.01, *n* = 6). Resistance values are expressed as percentage of initial resistance and compared to controls, respectively. For analysis of paracellular flux, controls were set to 100% and values were compared to this, respectively. All data shown are presented as means ± SEM.

To examine the effects of TNFα on the transepithelial barrier function of the porcine jejunal cell line IPEC-J2, concentration-dependent effects of the cytokine were analyzed by addition to the basolateral compartment of the cells, and TEER and [^3^H]-D-Mannitol flux were measured. After 48 h, 1000 U/mL and 5000 U/mL TNFα showed a marked decrease in TEER compared to the control group (one-way ANOVA: F (5,109) = 9.22, *p* < 0.0001, n = 14–22; ctrl: 101.06 ± 2.89%; 1000 U/mL: 88.45 ± 2.56%, *p* = 0.0113; 5000 U/mL: 78.74 ± 3.19%, *p* < 0.0001, Figure 1A). With lower concentrations of TNFα, TEER remained unchanged over the whole incubation period (50 U/mL: 97.47 ± 1.93%, *p* = 0.91; 100 U/mL: 102.21 ± 5.41%, *p* = 0.99; 500 U/mL: 98.49 ± 2.35%, *p* = 0.96). Hence, the following experiments were carried out using concentrations of 1000 U/mL TNFα. After 24 h, 1000 U/mL did not have significant effects on TEER (ctrl: 93.81 ± 1.81%; 1000 U/mL: 92.96 ± 3.60%, *p* = 0.99, *n* = 23; Figure 1B). 

For permeability studies, unidirectional paracellular tracer flux from the apical to the basolateral side of the cell culture filters was measured using [³H]-D-Mannitol. The control groups were set to 100% and flux measurements of TNFα-incubated cell filters were compared to control groups, respectively. For the first flux period of 24 h, 1000 U/mL TNFα indicated a stronger paracellular permeability compared to the control groups (ctrl: 100%; TNFα: 306.18 ± 59.76%, p = 0.006, n = 6). The same effect could be observed in the second flux period from 24 to 48 h (ctrl: 100%; TNFα: 256.32 ± 40.91%, p = 0.003, n = 6; Figure 1C).

### 2.2. Western Blot and Densitometry of TJ Proteins

**Figure 2:**
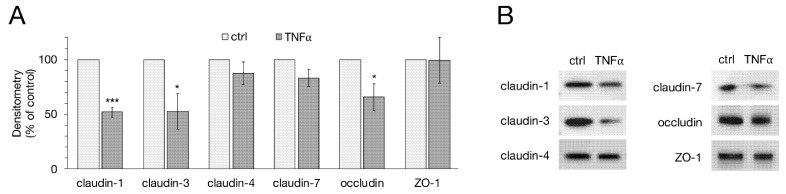
(**A**) Densitometry and (**B**) representative Western blot bands of tight junction (TJ) proteins after TNFα incubation revealed significantly weaker expression of claudin-1, -3 and occludin compared to controls, while claudin-4, -7 and ZO-1 remained unaffected (unpaired *t*-test, * *p* < 0.05, *** *p* < 0.001; *n* = 6). For Western blot analysis, the specific bands were normalized on total protein amount. All data presented are stated in means ± SEM.

Following electrophysiological measurements, protein samples of the cells were used for immunoblotting, analyzing TJ proteins claudin-1, -3, -4, -7, occludin and ZO-1. For densitometry, the protein levels of the specific bands were normalized on total protein amount. The protein expression of untreated cells was normalized to 100% and the TNFα-treated groups were compared to controls, respectively. Densitometric analysis of immunoblots revealed a significant decrease in claudin-1 expression in IPEC-J2 cells after treatment with TNFα for 48 h (51.94 ± 4.52%, *p* < 0.0001, *n* = 6). The same effect could be observed for claudin-3 (52.51 ± 16.09%, *p* = 0.012, *n* = 6), as well as for occludin expression (65.57 ± 12.31%, *p* = 0.019, *n* = 6). The expression of the TJ proteins claudin-4, -7 and ZO-1 did not significantly change during TNFα treatment (claudin-4: 87.39 ± 10.37%, *p* = 0.25; claudin-7: 82.99 ± 7.77%, *p* = 0.054; ZO-1: 99.36 ± 20.82%, *p* = 0.98; *n* = 6, Figure 2A,B).

### 2.3. Immunohistochemistry of TJ Proteins

**Figure 3:**
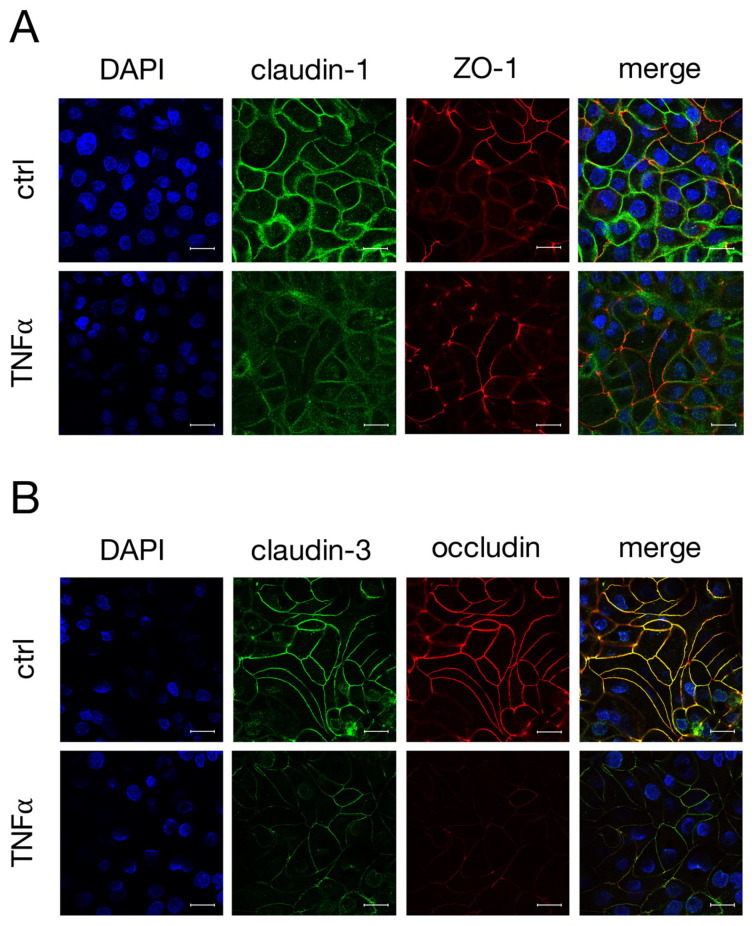
Confocal laser scanning immunofluorescence microscopy was performed to analyze the localization of (**A**) claudin-1 (green) and ZO-1 (red), or (**B**) claudin-3 (green) and occludin (red) after incubation with TNFα for 48 h. Nuclei were stained in blue (DAPI). Claudin-3 shows a strong colocalization with occludin (merge, yellow signal, B) (scale bar: 20 µm; *n* = 4; representative pictures).

As the incubation with TNFα led to changes in the expression levels of claudin-1, -3 and occludin in IPEC-J2 cells, confocal laser scanning immunofluorescence was performed to examine the localization of these TJ proteins. After 48 h incubation with TNFα, the claudin-1 signal (green, Figure 3A) no longer appeared as fine paracellular lines as seen for the controls, but rather as washed-out lines with a weaker signal. The ZO-1 signal remained mostly unaltered over the whole incubation period (red, Figure 3A). In Figure 3B, a co-localization of claudin-3 (green) and occludin (red), resulting in a yellow signal in merged confocal images, could be shown by double staining. TNFα caused an evident loss of specific claudin-3 and occludin immunofluorescence signals from the apicolateral membrane (Figure 3B), which correlates with the changes in the expression levels of claudin-3 and occludin.

### 2.4. TNFR-1 and TNFR-2 Expression Level

**Figure 4:**
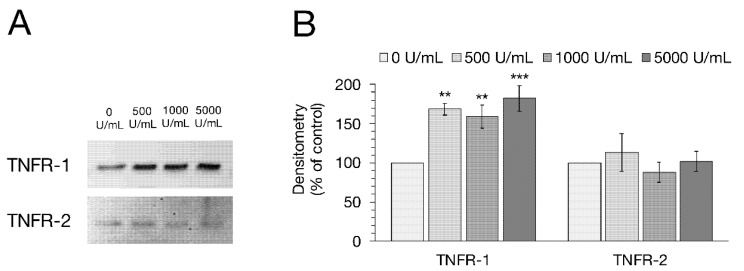
(**A**) Representative Western blot bands and (**B**) Densitometry of TNFR-1 and TNFR-2 after 48 h incubation with rising TNFα concentrations (500, 1000, 5000 U/mL). After specific protein bands were normalized on total protein amount, the protein expression of untreated cells was normalized to 100% and groups treated with TNFα were compared to this, respectively. Incubation with the cytokine led to a clear increase in TNFR-1 expression, while TNFR-2 remained unchanged. Data are shown in mean ± SEM (one-way ANOVA, ** *p* < 0.01, *** *p* < 0.001; *n* = 4).

Western blot analysis was performed to investigate whether the incubation with TNFα has any effect on the expression of TNFR-1 or -2. After normalization on total protein amount, the control groups were set to 100% and the TNFα-treated groups were compared to them, respectively. After 48 h with 500, 1000 and 5000 U/mL TNFα, a marked up-regulation of TNFR-1 expression could be observed (one-way ANOVA: F (3,12) = 9.70, *p* = 0.0016, *n* = 4; 500 U/mL: 168.34 ± 7.77%, *p* = 0.0036; 1000 U/mL: 159.08 ± 14.90%, *p* = 0.0098; 5000 U/mL: 182.35 ± 16.10%, *p* = 0.0008; Figure 4). Conversely, the densitometric analysis of TNFR-2 did not show any significant changes due to TNFα treatment (one-way ANOVA: F (3,12) = 0.46, *p* = 0.71, *n* = 4; 500 U/mL: 113.23 ± 24.21%, *p* = 0.87; 1000 U/mL: 88.00 ± 13.26%, *p* = 0.89; 5000 U/mL: 101.89 ± 12.57%, *p* = 0.99).

### 2.5. Confocal Laser Scanning Immunofluorescence Microscopy of TNFR-1

**Figure 5:**
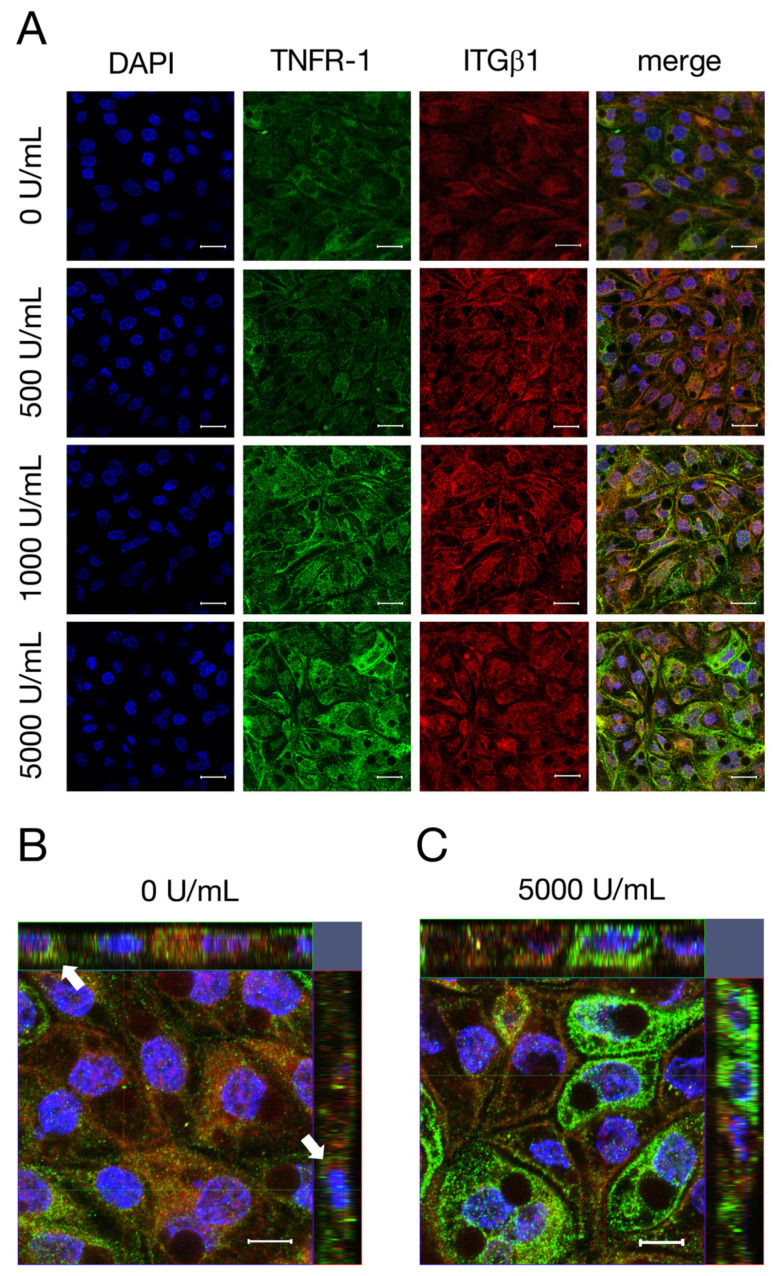
(**A**) To analyze the localization of TNFR-1 after incubation with different concentrations of TNFα (500, 1000, 5000 U/mL) for 48 h, confocal laser scanning immunofluorescence microscopy of TNFR-1 (green) and ITGβ1 (red, A) was performed. The colocalization of TNFR-1 with ITGβ1 can be seen as a yellow signal in the merged pictures. Nuclei were stained in blue (DAPI). In addition, Z-stack images with (**C**) or without (**B**) TNFα were recorded for a more detailed analysis of the localization of TNFR-1. (**B**) White arrows point out the basal side of the cells (scale bar: A 20 µm; B,C 40 µm; *n* = 3; representative images).

In addition to changes on the expression of TNFR-1, we aimed to analyze whether TNFα also leads to an altered localization of TNFR-1 in IPEC-J2. Therefore, cells were stained with TNFR-1 and ITGβ1, an integral membrane protein that is located at the basolateral membrane of cells [28]. With rising TNFα concentrations, a stronger signal for TNFR-1 (green, Figure 5A) could be observed, while the signal for ITGβ1 (red, Figure 5A) remained unchanged due to the incubation with TNFα. The co-localization of TNFR-1 with ITGβ1, resulting in the basolateral localization of TNFR-1, can be seen as a yellow signal in the merged pictures. Additionally, Z-stack images were performed to investigate the localization of TNFR-1 more precisely. In Figure 5B, the weak TNFR-1 signal (green) is solely orientated to the basal and basolateral membrane, which is pointed out by white arrows. After incubation with 5000 U/mL TNFα, the TNFR-1 signal appears entirely stronger and a localization, not only on the basolateral membranes of the cells, but also on the apical membrane, can be observed (Figure 5C).

### 2.6. Signaling Experiments with Specific MLCK Blocker

**Figure 6:**
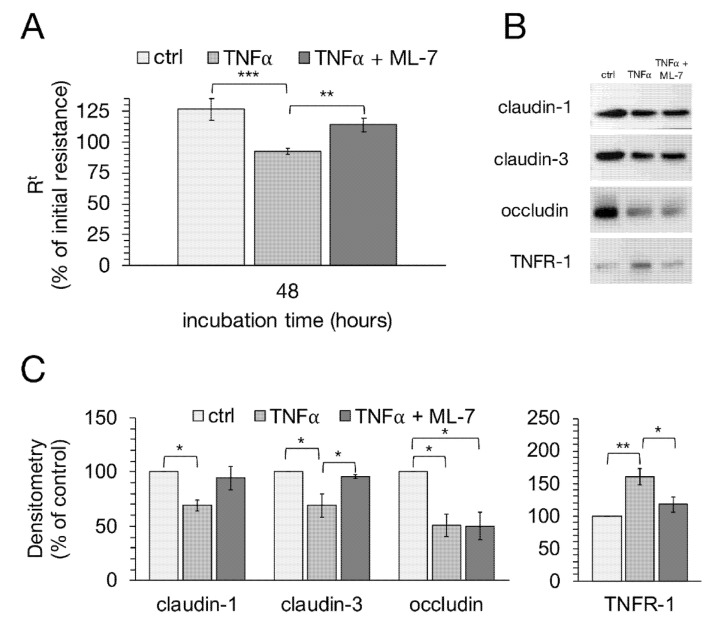
Effects of ML-7, a specific MLCK blocker, were studied after TNFα incubation using TEER measurements and Western blot analysis. (**A**) After 48 h, the groups treated solely with TNFα showed a significant difference in TEER compared to controls and cells treated with TNFα and ML-7 (one-way ANOVA, ** *p* < 0.01, *** *p* < 0.001; *n* = 7–29). (**B**) Representative Western blot bands and (**C**) densitometry after 48 h with TNFα in the presence or absence of ML-7 revealed that the MLCK blocker prevented the TNFα-induced decrease in claudin-1 and -3, while the decrease in occludin was not affected. Furthermore, the TNFα-induced increase in TNFR-1 is also inhibited by ML-7. Data are presented as mean ± SEM (* *p* < 0.05, ** *p* < 0.01; *n* = 4).

We used ML-7, a specific MLCK blocker, to analyze the signaling of TNFα-induced changes in epithelial barrier function in more detail. After 48 h, cells treated with TNFα together with ML-7 showed a significant difference compared to cells treated solely with TNFα (one-way ANOVA: F (2,41) = 15.37, *p* < 0.0001, *n* = 7–29; TNFα: 92.38 ± 2.66%; TNFα + ML-7: 113.76 ± 5.69%, *p* = 0.0053). Comparable results could be seen between cells treated with TNFα and control groups (TNFα: 92.38 ± 2.66%; ctrl: 126.50 ± 8.61%; *p* < 0.0001; Figure 6A). To investigate whether these changes are in connection with the altered expression of TJ proteins, immunoblot analysis was subsequently performed (Figure 6B,C). Densitometric analysis revealed that ML-7 almost completely averted the decrease in claudin-3 expression after treatment with TNFα (Kruskal–Wallis test: H (2) = 9.37, *p* = 0.009, *n* = 4; TNFα: 69.18 ± 10.86%; TNFα + ML-7: 95.58 ± 1.68%, *p* = 0.04). While the expression of claudin-1 was also notably higher with TNFα and ML-7 compared to cells treated only with TNFα, just a tendency towards significance could be shown (one-way ANOVA: F (2,9) = 5.30, *p* = 0.03, *n* = 4; TNFα: 69.34 ± 5.08%; TNFα + ML-7: 94.31 ± 11.17%, *p* = 0.079). Surprisingly, treatment with the MLCK blocker did not affect the expression of occludin (Kruskal–Wallis test: H (2) = 7.65, *p* = 0.02, *n* = 4; TNFα: 50.76 ± 10.04%; TNFα + ML-7: 50.28 ± 12.91%; *p* = 1.00). The strong increase in TNFR-1 due to TNFα incubation was also inhibited by ML-7 (Kruskal–Wallis test: H (2) = 9.09, *p* = 0.01, *n* = 4; TNFα: 160.70 ± 12.04%; TNFα + ML-7: 117.57 ± 11.32%; *p* = 0.026).

**Figure 7:**
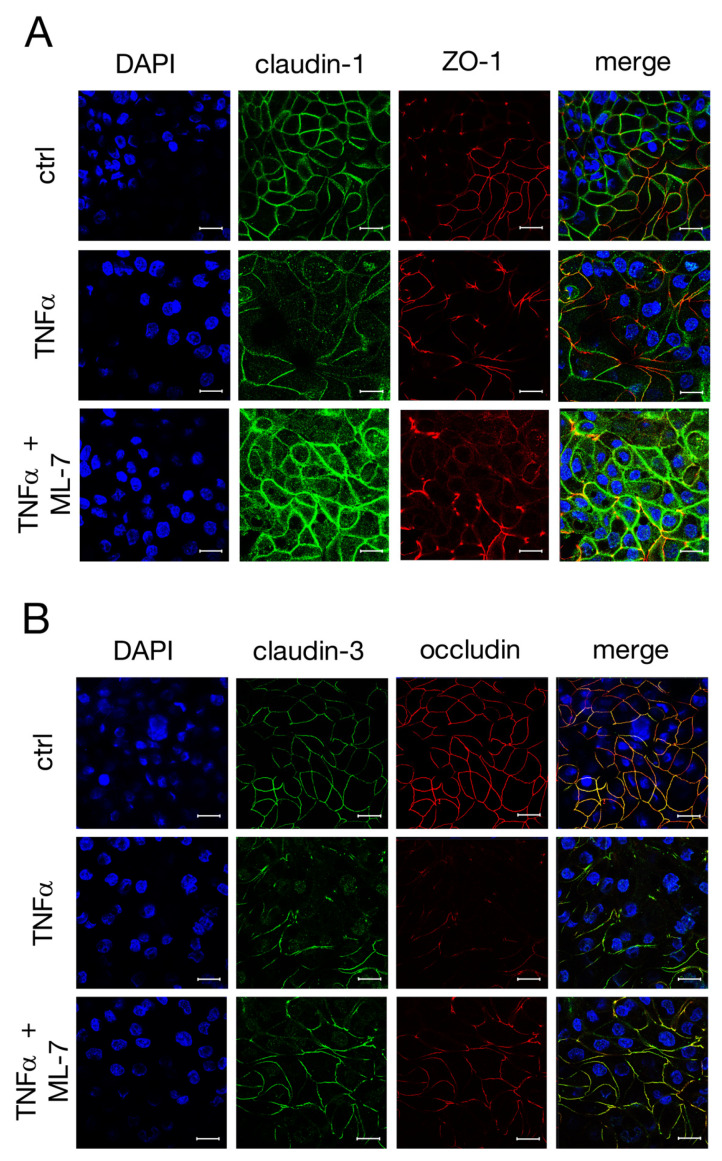
Confocal laser scanning immunofluorescence microscopy of (**A**) claudin-1 (green) and ZO-1 (red), as well as (**B**) claudin-3 (green) and occludin (red), nuclei were stained in blue (DAPI). IPEC-J2 monolayers were treated for 48 h with TNFα in the presence or absence of the specific MLCK blocker ML-7. (**B**) The yellow signal in the merged pictures represents the colocalization of claudin-3 and occludin (scale bar: 20 µm; *n* = 4; representative images).

Subsequently, confocal laser scanning immunofluorescence microscopy was carried out after 48 h to see whether ML-7 is also capable of inhibiting the changed localization of TJ proteins caused by TNFα. As shown in Figure 7A, the treatment with TNFα led to a loss of claudin-1 signal (green), in accordance with our earlier observations. ML-7 prevented the TNFα-induced changes since claudin-1 was detected as fine paracellular lines comparable to the controls. The paracellular signal of claudin-3 (green, Figure 7B) appeared to be mitigated compared to claudin-1 after treatment with the cytokine, while an increased intracellular signal was also observed. Furthermore, after TNFα-treatment, the occludin signal (red) appeared rather weak. Both can be partly prevented by ML-7, as claudin-3 and occludin were detected as fine, paracellular lines equivalent to the controls.

### 2.7. Recovery Experiment

**Figure 8:**
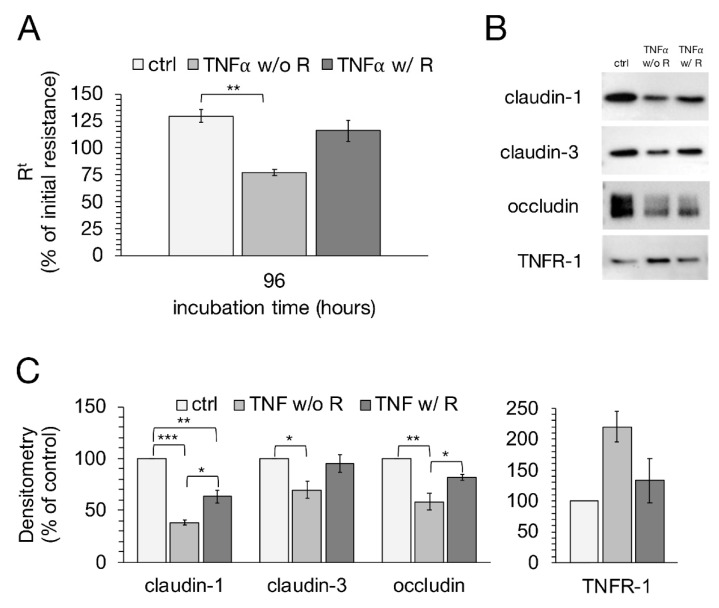
After IPEC-J2 cells were treated for 48 h with 1000 U/mL TNFα, a medium exchange was carried out and cells were either further incubated with the cytokine (TNFα without recovery; w/o R) or incubated with normal medium without TNFα (TNFα with recovery; w/R) for another 48 h. (**A**) TEER values of groups treated with TNFα removal were compared to cells treated without removal of the cytokine, respectively (one-way ANOVA, ** *p* < 0.01; *n* = 7–13). (**B**) Representative Western blot bands and (**C**) Densitometry of IPEC-J2 cells with or without recovery after 48 h incubation with TNFα revealed an increase in claudin-1 and occludin after removal of the cytokine, while claudin-3 expression did not show significant changes. The expression level of TNFR-1 also did not seem to be affected by TNFα removal. The shown data are presented as mean ± SEM (one-way ANOVA, * *p* < 0.05, ** *p* < 0.01, *** *p* < 0.001; *n* = 3).

Because TNFα has apoptotic potential [29,30], we performed further experiments to see whether IPEC-J2 cells can recover from incubation with the cytokine. Therefore, a medium exchange was carried out after 48 h, and cells were further incubated with or without TNFα. As shown in Figure 8A, cells with TNFα removal seem to recover from 48 h incubation with TNFα, since the TEER values showed a strong tendency to increase again, while the cells treated without cytokine removal decreased further on. However, the increase in TEER from cells with TNFα removal was not sufficient enough for a significant difference to the cells without the removal of the cytokine, but was close to being statistically significant (one-way ANOVA: F (2,26) = 6.64, *p* = 0.0047, *n* = 7–13; TNFα w/o Recovery: 77.36 ± 11.41%; TNFα w/ Recovery: 115.88 ± 15.65%; *p* = 0.076). The control groups showed a strong significant difference compared to the cells treated with TNFα for 96 h, (ctrl: 129.75 ± 7.82%; TNFα w/o Recovery: 77.36 ± 11.40; *p* = 0.0036). To analyze the effects a recovery period from TNFα might have on the changes in TJ proteins and TNFR-1, Western blot analysis and confocal laser scanning immunofluorescence microscopy were performed subsequently. A recovery period of 48 h after incubation with the cytokine led to a marked increase in claudin-1 expression in IPEC-J2 cells (one-way ANOVA: F (2,6) = 61.35, *p* = 0.0001, *n* = 3; TNFα w/o Recovery: 38.11 ± 2.34%; TNFα w/ Recovery: 63.20 ± 6.47%; *p* = 0.01; Figure 8B,C). Though, there was still a significant difference between controls and cells treated with TNFα recovery (Ctrl: 100%; TNFα w/ Recovery: 63.20 ± 6.47%; *p* = 0.002). Densitometric analysis of claudin-3 also revealed a rise after the removal of the cytokine, yet not to the same extent as claudin-1 (one-way ANOVA: F (2,6) = 5.84, *p* = 0.039, *n* = 3; TNFα w/o Recovery: 69.77 ± 8.10%; TNFα w/ Recovery: 95.08 ± 8.33%; *p* = 0.08). For occludin expression, the recovery period led to a strong increase compared to the cells treated with TNFα for 96 h (one-way ANOVA: F (2,6) = 16.24, *p* = 0.004, *n* = 3; TNFα w/o Recovery: 58.32 ± 8.40%; TNFα w/ Recovery: 81.70 ± 3.18%; *p* = 0.04). The increased expression of TNFR-1 due to TNFα incubation was not affected by the recovery period (Kruskal–Wallis test: H (2) = 4.78, *p* = 0.09, *n* = 3). 

**Figure 9:**
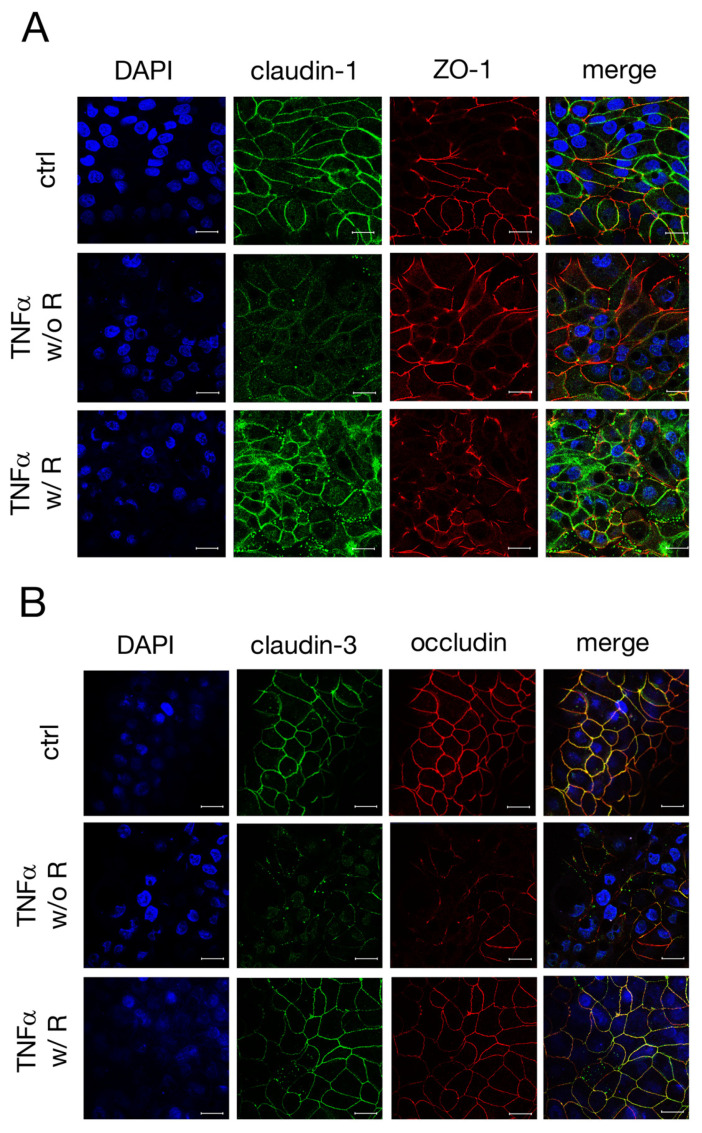
To analyze whether a recovery period from TNFα may have effects on the localization of changed TJ proteins due to treatment with the cytokine, confocal laser scanning immunofluorescence microscopy was performed subsequent to experiments. (**A**) Claudin-1 (green) and ZO-1 (red), and (**B**) claudin-3 (green) and occludin (red) were stained. Nuclei were stained in blue (DAPI). The yellow signal in the merged pictures constitutes a colocalization of the stained TJ proteins (scale bar: 20 µm; *n* = 3; representative pictures).

Moreover, confocal laser scanning immunofluorescence microscopy revealed that the paracellular TJ strands seemed to reconstitute after a recovery period of 48 h, as the claudin-1 signal appeared to be integrated again in the lateral membrane after a loss due to TNFα incubation (Figure 9A). Forty-eight hours after the removal of the cytokine, the signal for claudin-3 appeared to be as strong as detected in controls. The occludin signal also appeared to recover from the incubation with TNFα, but still showed a weaker signal compared to the controls, which was also reflected by a weaker yellow signal in the merged pictures (Figure 9B). Furthermore, a marked internalization of claudin-3 to sub-junctional compartments was observed when cells were incubated with TNFα for 96 h.

### 2.8. ApoTox-Glo Assay

In addition to the recovery experiments, an ApoTox-Glo assay was performed and the apoptotic potential of TNFα was further analyzed to ensure that the changes in barrier function are not due to the induction of apoptosis by the cytokine. Despite all this, neither the apoptotic or the cytotoxic rate nor the viability of IPEC-J2 cells was significantly changed after treatment with 1000 U/mL TNFα for 48 h. Hence, apoptosis could be eliminated as a cause for the decrease in transepithelial resistance in IPEC-J2 cells (data not shown).

## 3. Discussion

The intestinal mucosa provides one of the most important barriers to the outside environment, and an intact barrier is maintained by TJs linking adjacent epithelial cells and the immune system [31,32]. One of the main cytokines mediating between immune cells and barrier regulation is TNFα, a pro-inflammatory cytokine produced mainly by activated macrophages, monocytes and T-cells [33]. An increased, persistent production of TNFα has been shown to cause mucosal inflammation, leading to the destruction of the intestinal barrier with increased permeability due to the reduced function of the TJs, but also to the apoptosis of intestinal epithelial cells [34]. 

In our current work, we analyzed the effects of TNFα on the intestinal epithelial barrier function of the non-transformed porcine intestinal epithelial cell line IPEC-J2. Although this cell line has been described as a suitable in vitro model so far [19], the central effect of TNFα on the barrier function of IPEC-J2, in particular on the expression and localization of TJ proteins, has not been analyzed in detail yet. 

As already assumed, the use of TNFα showed a highly significant decrease in TEER, as well as an elevation in paracellular permeability to [³H]-D-Mannitol, both representing epithelial barrier function. To analyze this barrier-weakening effect more precisely, immunoblots and confocal laser scanning immunofluorescence microscopy of TJ proteins including claudin-1, -3, -4, -7, occludin and ZO-1 were subsequently carried out. In our study, strong yet differential effects of the cytokine on different TJ proteins were observed. Hence, the participation of the sealing TJ proteins claudin-1, as well as claudin-3 and occludin, on the disruption of epithelial barrier function could be shown, as the decrease in these integral membrane proteins after incubation with TNFα was in accordance with functional changes. In addition, not only a marked reduction in protein expression could be shown, but also a disruption of those TJ proteins, as the signal of claudin-1, -3 and occludin was either much reduced, disintegrated or located more intracellularly than in the cells treated without the cytokine. 

Because TNFα can bind two specific receptors, namely TNFR-1 and TNFR-2, we aimed to analyze if the observed changes are linked to the altered density of these receptors. Hence, we were able to detect a dose-dependent increase in TNFR-1 due to the incubation with TNFα, while TNFR-2 remained unchanged. One reason for this might be the expression of TNFR-1 in most tissues, while TNFR-2 appears to be found mostly in the lymphoid system. TNFR-1 is also the key signaling receptor for TNFα, as it is activated by both soluble and membrane-bound TNFα and mediates the signaling of apoptosis and inflammation [35,36]. Furthermore, TNFR-2 can only be fully activated by membrane-bound but not soluble TNFα [29].

To exclude the possibility that the observed changes are associated with an activation of apoptosis by TNFα [29,30], we performed recovery experiments as well as an ApoToxGlo assay. As the ApoTox-Glo assay did not show any significant changes in caspase-3/-7 activity, and the previously mentioned alterations in TEER and TJ proteins were reversible after the removal of the cytokine, we were able to eliminate apoptosis as a reason for the observed changes. 

Because the disruption of TJ proteins has been explained to be in accordance with an increased expression of MLCK [13,14,15,37], we analyzed if the TNFα-induced effects could be prevented by ML-7, a specific MLCK blocker [38,39]. TNFα can operate as an activator for MLCK, in turn leading to the increased permeability of the paracellular barrier [18]. Regarding a changed TJ barrier function, different routes can be involved, in particular the low-capacity leak and the high-capacity pore pathways [40,41]. The low-capacity leak pathway allows macromolecules to cross the TJ barrier, and is involved when TNFα stimulates MLCK [18]. To understand what role MLCK plays in our observed changes in TJ composition, cells were incubated with TNFα in the presence or absence of ML-7. TEER was recorded and the protein expression and distribution of claudin-1, -3, occludin and TNFR-1 were analyzed, indicating MLCK as the major pathway component of TNFα signaling. 

Incubation with TNFα in the presence of the specific MLCK blocker ML-7 prevented the decrease in TEER and claudin-3 expression. However, in our experiments, occludin expression was not significantly altered. Whether the effects of MLCK are related to a role for myosin transport of the affected proteins, or if MLCK might have other, e.g., structural roles [42,43], could not be addressed within the scope of the current study in more detail, but might be of interest for ongoing research. However, as a possible major MLCK substrate, myosin light chain II (MLC-2) has been shown to mediate alterations of paracellular permeability in gastrointestinal disorders [14].

In our current study, the pharmacological perturbation of MLCK was performed by the small molecule inhibitor ML-7. Although additional results such as (i) MLCK knockdown during TNFα treatment and (ii) MLCK overexpression in the absence of TNFα might even have strengthened the study, the blocking experiment has proven reliable for a convincing conclusion on the role of MLCK. Other additional mechanisms and signaling events might be still involved, though.

In a publication from Xiao et al., IPEC-J2 cells were stimulated with TNFα to analyze whether TGF-β1 may have protective effects. After TNFα challenge, a reduction in occludin and ZO-1 of IPEC-J2 monolayers was observed, while the TJ protein claudin-1 remained unaffected [44]. This is in contrast with the alteration of TJ proteins in our work, as in our experiments, the TJ proteins claudin-1, -3 and occludin were markedly reduced after incubation with TNFα. Thus, our findings demonstrate once again that the cell line IPEC-J2, cultured under species-specific conditions using porcine serum instead of conventional serum [20], shows an improved model for the analysis of porcine jejunal epithelium, as the changes in TJ composition are more commensurate to those in other models after TNFα challenge [45,46].

An effect of TNFα not only on sealing but also on pore-forming tight junction proteins has been described previously [11]. In different models [47,48,49], incubation with TNFα led to a significant increase in claudin-2, a paracellular channel for small cations and water [50]. Due to the lack of IPEC-J2 regarding the expression of pore-forming TJ proteins [20], an examination of the effect on these particular proteins could not be performed in our current approach. However, with a lack of claudin-2 expression, the TJ expression pattern of IPEC-J2 appears to be very close to Peyer’s patches follicle-associated epithelium, therefore representing the main relevant epithelium involved in immune surveillance in the intestine [51,52]. Currently, many studies demonstrate the susceptibility and reliability of the non-malignant porcine epithelial cell line IPEC-J2 regarding a wide variety of mechanisms in native tissue of different species in vitro [19,53].

## 4. Material and Methods

### 4.1. Cell Culturing and TNFα Treatment

Confluent monolayers of the porcine jejunal cell line IPEC-J2 (DSMZ, Braunschweig, Germany) were grown in 25 cm^2^ culture flasks in Dulbecco’s MEM/Ham’s F-12 (Biochrom, Berlin, Germany) containing 3.15 g/L glucose, 2 mM stable glutamine, 10% porcine serum (Sigma Aldrich, Munich, Germany) and 1% penicillin/streptomycin (Sigma Aldrich, Munich, Germany). Cells were cultured at 37 °C in a humified 5% CO_2_ atmosphere. The medium was changed every 2–3 days and cells were split once a week at a ratio of 1:3. For electrophysiological measurements, cells were seeded at a density of 2 × 10^5^ cells/mL on semipermeable cell culture inserts with a diameter of 12 mm and a pore size of 0.45 µm (Millipore, Darmstadt, Germany) and placed into multi-well plates. Routinely, 500 µL of media was added to the apical compartment, and the basolateral compartment was filled with 1 mL of media to guarantee an equal hydrostatic pressure, as specified by the manufacturer. Experiments were performed after cells reached similar R^t^ values (~14 days) to ensure a functional barrier. Therefore, recombinant human TNFα (peprotech, Hamburg, Germany) was added in various concentrations (50, 100, 500, 1000 and 5000 U/mL) to the basolateral side of the cell culture inserts, and transepithelial resistance was monitored for 48 h. The cells were further processed and used for immunoblotting and immunohistochemistry, as described below. Resistance values were corrected with the blank values of the filters and the media used in the experiments. Only cells with passages between 7 and 12 were used for experimental purposes.

### 4.2. TEER and Flux Measurements

Transepithelial electrical resistance (TEER) measurements, presenting the epithelial barrier function, were assessed by using a chopstick electrode and an Epithelial Volt/Ohm Meter (EVOM, World Precision Instruments, Sarasota, FL, USA). Permeability studies were carried out using [³H]-D-Mannitol to examine the unidirectional paracellular tracer flux from the apical to the basolateral side of the cell filters. Cells were seeded on semipermeable cell culture inserts as described previously. A total of 0.18 µCi of [³H]-D-Mannitol (PerkinElmer, Waltham, MA, USA) was added to the apical side of the filters, and samples of 50 µL were taken directly and 48 h after addition of the tracer. Subsequently, the specific activity of the tracer was calculated using Equation (1).

(1) $$\mathrm{specific}\;\mathrm{activity}\;\left[\mathrm{nmol}\right]=\frac{\mathrm{mean}\left({\mathrm{counts}}_{\mathrm{donor}\;\mathrm{side}}\right)}{{\mathrm{concentration}}_{\mathrm{donor}\;\mathrm{side}}\times {\mathrm{volume}}_{\mathrm{donor}\;\mathrm{side}}}$$

For permeability measurements, samples of 300 µL were taken every 24 h during the incubation with the cytokine from the basolateral side, resulting in two flux periods. The removed media were replaced with fresh media containing the corresponding TNFα concentration. Following the sampling, Aquasafe 300plus liquid scintillation cocktail (Zinsser Analytic, Frankfurt, Germany) was added and each sample was examined using TriCarb 4910TR liquid scintillation counter (PerkinElmer, Waltham, MA, USA). By using Equation (2), the resulting paracellular flux was calculated.

(2) $$J\left[\mathrm{nmol}\;\times {\;\mathrm{cm}}^{-2}\;\times {\;h}^{-1}\right]=\;\frac{{\mathrm{counts}}_{t}\;\times \;\frac{V_{\mathrm{chamber}}}{V_{\mathrm{sample}}}-{\;\mathrm{counts}}_{t-1}\times \frac{V_{\mathrm{chamber}}-{\;V}_{\mathrm{dilution}}}{V_{\mathrm{sample}}}}{\mathrm{specific}\;\mathrm{activity}\;\times \;\mathrm{area}\times \mathrm{time}}\;$$

### 4.3. Protein Extraction and Quantification

After incubation of the cells with TNFα, IPEC-J2 monolayers were washed in PBS with calcium and magnesium and lysed in RIPA buffer, containing 25 µM HEPES pH 7.6, 25 µM NaF, 2 µM EDTA, 1% Sodium Dodecyl Sulfate (10%), H2O and enzymatic protease inhibitors (Complete EDTA-free, Boehringer, Mannheim, Germany). Cells were then scraped off the permeable supports and the suspension was transferred into Eppendorf tubes. Samples were homogenized after incubation on ice for 30 min. Protein quantification was performed by using Bio-Rad DC Protein Assay (Bio-Rad Laboratories GmbH, Munich, Germany) as prescribed by the manufacturer. For the detection, EnSpire Multimode Plate Reader (Perkin Elmer, Waltham, MA, USA) was used.

### 4.4. Immunoblotting and Densitometry

Western blot analysis was performed to identify the TJ protein expression after TNFα treatment. Protein (20 µg) and Laemmli buffer (Bio-Rad Laboratories GmbH, Munich, Germany) were mixed and loaded onto 10% TGX Stain-Free FastCast gels (Bio-Rad Laboratories GmbH, Munich, Germany). Electrophoresis was carried out for 60 min at 150 V. Subsequently, samples were transferred onto a PVDF membrane for 90 min at 100 V and blocked for 60 min in 5% milk (in Tris-buffered saline with 0.1% Tween 20). Membranes were then incubated with specific antibodies raised against TJ proteins (all from Thermo Fisher Scientific) claudin-1 (cat. #51-9000), -3 (cat. #34-1700), -4 (cat. #32-9400), -7 (cat. #34-9100), occludin (cat. #33-1500) and ZO-1 (cat. #33-9100) following the manufacturer’s instructions at 4 °C overnight, respectively. To bind the primary antibodies, horseradish-peroxidase-conjugated goat anti-mouse or goat anti-rabbit antibodies were used for 1 h at room temperature. Specificity was shown in detail previously [19,51,52]. For visualization of the protein bands, Clarity Western ECL Blotting Substrate (Bio-Rad Laboratories GmbH, Munich, Germany) was used after the total protein amount was detected with the ChemiDoc MP Luminescence imager (ChemiDoc MP, Munich, Germany). Later, the density of the specific bands was quantified and analyzed using the imager-associated software Image Lab. For densitometry, bands were normalized on total protein amount and compared to the control groups, respectively.

### 4.5. Immunohistochemistry

Confocal laser scanning immunofluorescence microscopy was performed for the detection and localization of TJ proteins after TNFα treatment. Therefore, cells were washed twice with PBS and fixated in ice-cold methanol for 10 min at −20 °C. Permeabilization was carried out using Triton X-100 for 10 min at room temperature. Cells were then blocked for 60 min in PBS containing 1% bovine serum albumin and 5% goat serum and subsequently incubated with primary antibodies raised against claudin-1, -3, occludin and ZO-1 for 60 min at 37 °C (Thermo Fisher Scientific; claudin-1 (cat. #51-9000), -3 (cat. #34-1700), occludin (cat. #33-1500) and ZO-1 (cat. #33-9100)). Again, cells were washed with PBS and then incubated with secondary goat anti-rabbit Alexa Fluor-488 (1:1000, Thermo Fisher Scientific, cat. #A-11008) or goat anti-mouse Alexa Fluor-594 (cat. #A-11005) for 60 min at 37 °C. Nuclei were stained with DAPI (1:2000) for 5 min at room temperature. Following this, filters were mounted with ProTaqs Mount Fluor (Biocyc, Luckenwalde, Germany) and slides were analyzed by using Zeiss 710 confocal microscope (Zeiss, Oberkochen, Germany).

### 4.6. Investigation of Dose-Dependent Changes in the Expression Level and Localization of Tumor Necrosis Factor Receptor 1 or 2 after Treatment with TNFα

To examine whether the treatment with TNFα affects the expression level of tumor necrosis factor receptors 1 and 2 (TNFR-1, TNFR-2) in IPEC-J2 cells, Western blot analysis was carried out as mentioned above. Membranes were incubated with rabbit polyclonal antibodies raised against TNFR-1 (abcam, cat. #ab1939) and TNFR-2 (antibodies-online, cat. #ABIN2789622). After detection, specific bands were normalized on total protein amount and analyzed compared to the untreated groups, respectively. Moreover, immunohistochemistry of IPEC-J2 cells, treated with rising TNFα concentrations, was performed for examination of expression level and localization of TNFR-1. Therefore, a staining using specific antibodies raised against TNFR-1 together with a marker for the basolateral membrane, namely Integrin beta-1 (ITGβ1; Thermo Fisher, Rockford, IL, USA), was carried out as mentioned above. To analyze the location of TNFR-1 in more detail, Z-stacks were performed additionally. 

### 4.7. Specific Myosin Light Chain Kinase (MLCK) Blocker in TNFα-Induced Changes in Epithelial Barrier Function

For a more detailed characterization of the signaling in TNFα-induced barrier changes, ML-7 (Sigma Aldrich, Munich, Germany), a specific blocker against MLCK, was used. Therefore, IPEC-J2 cells were incubated with TNFα (1000 U/mL) in the presence or absence of ML-7 (10 µM). Stock solutions of ML-7, dissolved in dimethyl sulfoxide (DMSO; Sigma Aldrich, Munich, Germany), were diluted in medium and added simultaneously with TNFα to the apical and basolateral compartment of the cell filters. Respectively, 0.1% of DMSO was added to controls as well as to cell filters incubated with TNFα without ML-7, to exclude a DMSO-dependent effect on IPEC-J2 cells. TEER measurements were carried out before addition and after 48 h. Subsequent to TEER measurements, cells were either fixated for immunohistochemistry or proteins were extracted and further processed for immunoblotting, analyzing localization and expression of TJ proteins after experiments with or without ML-7. 

### 4.8. Recovery Experiments in IPEC-J2 after Removal of TNFα

Because TNFα has been described to have apoptotic potential, we wanted to investigate if the IPEC-J2 cells can recover from the TEER decrease after a TNFα-challenge. Therefore, cells were incubated with 1000 U/mL TNFα for 48 h as described above. Subsequently, a medium exchange was carried out and fresh media without TNFα were added to the cells that were treated with the cytokine before. Following TEER measurements, possible alterations of TJ proteins were examined using Western blot technique or immunohistochemistry as described above.

### 4.9. ApoTox-Glo Assay

IPEC-J2 cells were seeded as triplicates at a density of 2 × 10^5^ cells/mL on PET cell culture inserts of a 24-well Transwell system with 6.5 mm membrane diameter and a 0.4 µm pore size (Costar, Corning Incorporated, Kennebunk, ME, USA). After a cultivation period of 14 to 16 days, cells were treated with TNFα (1000 U/mL) and further incubated for 48 h. To measure the viability, cytotoxicity and apoptosis of the IPEC-J2 cells, the ApoTox-Glo^TM^ Triplex Assay (Promega GmbH, Walldorf, Germany) was carried out according to the manufacturer’s specifications. After the incubation period with TNFα, the viability/cytotoxicity reagent, containing both the bis-AAF-R110 substrate and GF-AFC substrate, was added to each well and mixed for 30 s on an orbital shaker. After an incubation of 30 min at 37 °C, the fluorescence was measured at 400EX/505EM for viability and 485EX/520EM for cytotoxicity. Following this, the apoptosis reagent, containing the Caspase-Glo 3/7 substrate, was added to each well, mixed gently and incubated for another 30 min at constant room temperature. Luminescence was measured to detect cell apoptosis. All measurements were carried out using EnSpire Multimode Plate Reader (Perkin Elmer, Waltham, MA, USA).

### 4.10. Statistical Analysis

Data are always expressed in means and standard error of the mean (SEM). N represents the number of cell culture inserts used unless stated otherwise. For TEER measurements, statistical analysis was performed using one-way ANOVA and Dunnett’s test for the correction of multiple testing. For comparison between two groups, unpaired Student’s *t*-test was used. Statistical analysis for densitometry of Western blots was performed with one-way ANOVA for normally distributed data and with Kruskal–Wallis test for non-normally distributed data. For the signaling experiments, Tukey–Kramer method was used as post hoc test for pairwise comparisons. Values of *p* < 0.05 were considered to be statistically significant, being presented as * *p* < 0.05, ** *p* < 0.01 and *** *p* < 0.001. 

## 5. Conclusions

Employing the non-transformed intestinal epithelial cell line IPEC-J2, our study demonstrates, for the first time, that the exponential nature of barrier impairment by TNFα in porcine intestinal inflammatory processes can be explained by the finding that its own receptor, TNFR-1, is upregulated by TNFα itself. 

## Data Availability

Data are contained within the article. The datasets analyzed during the current study are available from the corresponding author upon reasonable request.
